# Fellowships, Grants, & Awards

**Published:** 2004-09

## Understanding and Promoting Health Literacy

The participating institutes, centers, and offices of the NIH and the Agency for Healthcare Research and Quality (AHRQ) invite investigators to submit research grant applications on health literacy. The goal of this program announcement (PA) is to increase scientific understanding of the nature of health literacy and its relationship to healthy behaviors, illness prevention and treatment, chronic disease management, health disparities, risk assessment of environmental factors, and health outcomes including mental and oral health. There is a need for increased scientific knowledge of interventions that can strengthen health literacy and improve the positive health impacts of communications between health care/public health professionals (including dentists, health care delivery organizations, and public health entities) and consumer or patient audiences that vary in health literacy. Such knowledge will help enable health care and public health systems to serve individuals and populations more effectively, and employ strategies that reduce health disparities in the population. Once a general understanding of the various factors influencing current trends has been achieved, a number of secondary goals may be addressed. Applicants may propose secondary goals of modeling the potential impact of new interventions on future national trends and/or determining the impact of targeted cancer control interventions on population outcome (i.e., evaluating optimal cancer control strategies).

Healthy People 2010 defines “health literacy” as the degree to which individuals have the capacity to obtain, process, and understand basic health information and services needed to make appropriate health decisions. Many factors affect individuals’ ability to comprehend, and in turn use or act on, health information and communication. Proficiency in reading, writing, listening, interpreting, oral communication, and visual analysis is necessary as the modern health system typically relies on a variety of interpersonal, textual, and electronic media to present health information. Individuals and families both must be able to 1) communicate with health professionals; 2) understand the health information in mass communication; 3) understand how to use health-related print, audiovisual, graphic, and electronic materials; 4) understand basic health concepts (e.g., that many health problems can be prevented or minimized) and vocabulary (e.g., about the body, diseases, medical treatments, etc.); and 5) connect this health-related knowledge to health decision making and action taking.

Access to and understanding of health information and services is a reciprocal process among health professionals, communication professionals, and patients. For instance, these professionals must use science-based strategies and tactics, develop resources and materials, and understand communication interactions between providers and patients.

Research on health literacy should assist the NIH in its mission of communicating scientifically based health information to the public and to the health care providers and related professionals who serve the public. The application of scientific knowledge from health literacy research may also strengthen the health information knowledge and communication skills of the public, and further one of the national goals of Healthy People 2010: to improve health literacy by the decade’s end.

Health literacy is a complex phenomenon that involves individuals, families, communities, and systems. For instance, consumers, patients, caregivers, and other laypersons may vary with respect to 1) access (e.g., to audience-appropriate information, media, or professionals); 2) skills (e.g., to gather and comprehend health information; to speak and share personal information about health history and symptoms; to act on information by initiating appropriate follow-up visits and conveying understanding back to the information source; to make decisions about basic healthy behaviors, such as healthy eating and exercise; to engage in self-care and chronic disease management); 3) knowledge (e.g., of health and medical vocabulary, concepts such as “risk,” the organization and functioning of health care systems); 4) disabilities (e.g., sensory, communication, cognitive, or physical challenges or limitations); 5) the features of their health care providers and the public health systems in which these providers practice (e.g., the communication skills of health professionals, platforms employed for patient education, built environments and signage); or 6) other important characteristics (including development or life stage; cultural, linguistic, or educational differences that affect health beliefs, knowledge, or communication).

Too often, people with the greatest health burdens have limited access to relevant health information. In part, this is due to the complex and cumbersome ways in which health information often is presented; it is also due to individuals’ limited abilities to fully interpret and understand complex health terminology and instructions, and to make personal decisions related to risk-avoidance or -reduction strategies. For instance, to follow health care instructions, patients need to be able to comprehend written and oral prescription instructions, directions for self-care, and plans for follow-up tests and appointments.

In addition, health care providers may not communicate effectively with individuals with limited levels of literacy. For instance, achieving informed consent for treatment is difficult when health care personnel cannot explain biological processes or treatment procedures in simplified language, and patients cannot interpret health information. These situations hamper the effectiveness of health professionals’ efforts to prevent, diagnose, and treat medical conditions, and limit many health care consumers’ abilities to make important health care decisions.

Low health literacy is a widespread problem, affecting more than 90 million adults in the United States. Low health literacy results in patients’ inadequate engagement in and benefit from health care advances, as well as medical errors. Low health literacy is likely to be a major contributor of adverse health outcomes. Research has linked low or limited health literacy with such adverse outcomes as poorer self-management of chronic diseases, less healthful behaviors, higher rates of hospitalizations, and overall poorer health.

This PA invites applications to develop research on health literacy in general areas that include, but are not limited to, the following: 1) modeling and measuring the nature and scope of health literacy; 2) variation in health literacy over the life course or among native and nonnative speakers of English; 3) mediators and moderators of low health literacy; 4) the impact of low health literacy on health outcomes, diseases, behaviors, and treatments, including the contribution of health literacy to informed decision making, adherence to preventative or therapeutic regimens, utilization of health care services, risk avoidance strategies, and other consumer health care–related actions; 5) the identification of effective preventive and other interventions to improve health literacy among populations and to enable the health care and public health systems to communicate effectively across different health literacy levels; and 6) the development of effective methods and new technologies in health literacy research.

Applications should be relevant both to the objectives of the PA and to at least one of the participating institutes’ general research interests. Prior to preparing an application, researchers are strongly encouraged both to review the general research interests of the participating institutes and to contact program staff of the relevant institutes to discuss the proposed research.

A wide variety of research approaches are encouraged under this PA: basic research that investigates or describes the nature of health literacy and the magnitude of health literacy problems, and applied research addressing issues pertinent to health literacy practices (e.g., systems-level interventions) and research in practice (e.g., active potential end users participate as supportive research partners). Applications also may develop theoretical models, refine research constructs, improve methods and measurements, and establish causal relationships (e.g., between low health literacy and lack of effective health promotion). Researchers also may address the effectiveness of interventions, or adapt and test existing programs (including those that are not research-based) to reduce low health literacy and its adverse consequences (e.g., interventions implemented by health care systems and systems outside of health care such as systems of public education).

The research must involve either 1) health literacy, or one of its many components, as a key outcome; 2) health literacy as a key explanatory variable for some other outcome; 3) methodological or technological improvement to strengthen research on health literacy; or 4) health literacy–focused preventions and interventions. Studies to develop or evaluate the readability or utility of specific materials that are intended for single uses or single audiences are not responsive to this PA unless these investigations are integral to testing a significant research hypothesis related to health literacy. Some potential areas of focus are as follows.

### Nature and scope.

1) Assess the prevalence and causes of low health literacy; 2) identify the nature of the mix of abilities and skills required to be functionally “health literate” (e.g., including media and health care system navigation skills, etc.) and the roles of basic literacy (i.e., reading, writing, speaking, listening, visual interpretation skills) and mathematics abilities (e.g., graphical interpretation and other quantitative skills) in health literacy; 3) explore the magnitude and variation, by socioeconomic and/or other group characteristics, in accessing, seeking, evaluating, interpreting, and using health information from a variety of sources; 4) examine the problems and factors involved in the presentation and interpretation of quantitative information (e.g., graphic interpretation, “risk” or probability statistics, the influence of information context and information formats, etc.) from either the provider or user perspective, or investigate how specific health referents, such as basic genetics and/or environmental risk concepts, are best understood and conveyed; 5) create a conceptual model of “health literacy” or the skill sets that influence the comprehension of relevant health information (e.g., visual information comprehension skills that permit understanding of such visual messages as color coding, representation of risk, or disease processes); or 6) evaluate the different strategies and channels available, including the role of information technologies, that enable consumers to seek, access, and interpret relevant health information effectively, and how these may differ by cultural and health literacy backgrounds (e.g., research on the information-seeking or service-utilization characteristics among health consumers with different levels of health literacy).

### Life span and cultural differences.

Applications addressing health literacy as an age-differentiated phenomenon might explore the developmental precursors of low health literacy and the age-related changes in reading and other cognitive skills throughout the life course that may contribute to these difficulties. 1) Identify the reading and oral language comprehension skills crucial for the satisfactory acquisition and understanding of basic health information by children, adolescents, and adults of various ages; 2) determine how intuitive or everyday notions of germs, contagion, environmental exposures, disease, drugs, bodily processes, and other health-related concepts influence health literacy and consequent illness prevention behaviors across the life course, and identify age-appropriate intervention techniques that can be used to mitigate these problems; 3) examine the role of social and cultural factors in the development of health literacy (e.g., how children acquire health-related knowledge as they age, especially those children in households where the parents speak limited English and the children serve as interpreters); 4) explore how the quantity and quality of structured interactions with adult caregivers affects the health literacy of the child from birth to age three; or 5) examine the effect of current age-related differences in media use (e.g., children versus the elderly) on health literacy.

### Mediators and moderators of health literacy: protective and risk factors.

1) Describe how patients’ information-seeking abilities and health information interpretation mediates or moderates the effects of provider practices on health literacy; 2) examine bidirectional communication processes between providers and patients/clients in the health care/health promotion system that affect health literacy, including systemic and cultural barriers that help create and sustain health literacy problems, as well as adaptation strategies used by providers and consumers to minimize health literacy problems (e.g., how patients’ use of print and electronic health information mediates or moderates their communication with providers); 3) examine how physicians’ or dentists’ nonverbal communication influences patients’ comprehension and implementation of health-related information; 4) examine the influence of social, contextual, and environmental factors (e.g., urban versus rural, housing type, workplace features, social support and social network members, etc.) on health literacy outcomes; 5) examine the media (including TV, radio, movies, newspapers, the Internet, and interactive systems) as a socializing agent of health literacy (e.g., determine how newspaper articles, TV drug advertising, soap operas, and medical dramas affect health literacy, and how different media can be used to communicate more effectively with consumers varying in health literacy levels); or 6) examine the factors that influence the desire for or processing of health literacy information (e.g., how self-efficacy in decision making and/or financial planning, time perspectives as presented in socio-emotional selectivity theory, ease of cognitive access via intuitive and reasoning processes, and coping and anxiety reduction behaviors influence the use of or desire to access health care knowledge).

### Impacts and consequences of low health literacy.

1) Examine the relationship between health literacy and health disparities; 2) analyze the role of health literacy in the prevention and treatment of chronic diseases; 3) identify the relationship between health literacy variation and the ability to engage in informed decision making for a variety of health issues, such as chronic disease management and participation in clinical trials; 4) evaluate the magnitude of the problems caused by low levels of health literacy or by professionals’ lack of effective communication skills for adapting to the communication needs of consumers with differing levels of literacy; or 5) assess the role of health literacy as a mediator or moderator of health care access across adulthood.

### Preventative interventions: education and training.

1) Explore the role of kindergarten through twelfth-grade (K–12) education systems in increasing levels of health literacy and improving health communication skills (e.g., assess the treatment of health literacy in K–12 health education, biology, or general science classes; assess the effectiveness of such course-work, curricula, and pedagogy on improving health literacy among school-age children; evaluate the effectiveness of arts-based interventions on children’s development of health literacy); 2) assess the role of K–12 education (e.g., in basic literacy skills such as reading, writing, comprehension, speaking, and listening skills, or in mathematics) on health literacy; or 3) determine the specific content and components of undergraduate, graduate, and in-service training experiences needed to adequately prepare provider groups to communicate with low-literacy patient populations (e.g., assess the effect of cultural competence on provider communication skills; assess innovative training approaches that allow providers to help patients deal with shame over low health literacy and facilitate negotiating the modern health care system); examine policies that support the development, implementation, and effectiveness of such training experiences; and evaluate the roles of information technology in training to improve health literacy.

### Other health literacy interventions.

1) Evaluate the effectiveness of health literacy interventions directed at the general public, different audience segments, patients, providers, or the health care or public health systems (e.g., how health care systems can be designed to better support the information needs of consumers with different levels of health literacy; the effectiveness of interventions within the health care system that are designed to increase the access of intended audiences to relevant health information and appropriate materials); 2) examine the development and dissemination of effective information sources and materials for audiences with different levels of health literacy (e.g., how prevention campaigns should be designed to effectively communicate with audiences with differing levels of health literacy); 3) design and evaluate health literacy diagnostic and/or communication tools to help health care professionals identify and communicate more effectively with consumers with different levels of health literacy (e.g., technology tools for automatically converting health information to a variety of appropriate levels); 4) identify innovative strategies, practices, and policies currently in use that can be disseminated immediately to promote health literacy across the various participants in the health care systems; 5) conduct cost-effectiveness analyses of various health literacy interventions; or 6) further multilevel health literacy intervention approaches (e.g., by developing paradigms and/or statistical models to test the interaction of such variables as knowledge, prior education, cognitive status, social support, community influence, technology, and health care access on health care decisions).

### Methodology and research technology development.

1) Assess the efficacy of current methods of health literacy assessment and develop, as needed, audience-appropriate methodologies to understand the prevalence of low health literacy in different populations, the interaction of low health literacy with other demographic and social factors, and the contribution of low health literacy to health care costs and health outcomes; 2) identify effective approaches of combining qualitative and quantitative methods to further knowledge of health literacy; 3) identify a core set of constructs, variables, and quantitative measures for conducting health literacy research; 4) develop and pilot-test new tools and technologies to identify health literacy barriers (e.g., an assessment to distinguish, among persons with low literacy skills, those who have learning disabilities or communication disorders such as auditory processing, aphasia, or hearing loss); or 5) in the context of understanding and promoting health literacy, develop technologies related to data reduction, data mining, and knowledge extraction; develop tools for meta-databases and integrative services to enhance the utility of existing databases; or develop new methods or technologies for timely, appropriate communication of pertinent health information and knowledge (e.g., as seen through the creation of telemedicine, or to enhance patient, doctor, or administrator decision making regarding health literacy, etc.).

Projects may employ any one or combination of study designs, research approaches, and data collection techniques. Secondary analyses of existing data sets as well as meta-analytic studies are also suitable for this PA. Multilevel, multidisciplinary, and interdisciplinary research is also encouraged, especially studies that incorporate individual, family, community, and societal mediators of health literacy in childhood and adulthood, or state-of-the-art health communication theory and knowledge. Researchers are encouraged to address ongoing investigations of prevention, healthy living, chronic disease management, patient-based health care, cultural competence, and health disparities to inform the research on health literacy. Research questions can focus on consumers, patients, clients, or other population groups; the strategies and tactics used by providers of medical and health information/communication to enable them to effectively reach literacy-challenged populations; or the influences of health literacy upon interactions between consumers, patients, clients, providers, and organizations or systems.

The Institute of Medicine’s 2004 report *Health Literacy: A Prescription to End Confusion* reviews the current body of knowledge about health literacy, and identifies actions for the promotion of health literacy in society. Applicants are encouraged to consult this report as a general reference.

This PA will use the NIH R01 award mechanism. As an applicant, you will be solely responsible for planning, directing, and executing the proposed project. This PA uses just-in-time concepts. It also uses the modular budgeting format (see http://grants.nih.gov/grants/funding/modular/modular.htm). Specifically, if you are submitting an application with direct costs in each year of $250,000 or less, use the modular budget format. This program does not require cost sharing as defined in the current NIH Grants Policy Statement at http://grants.nih.gov/grants/policy/nihgps_2003/NIHGPS_Part2.htm.

Applications must be prepared using the PHS 398 research grant application instructions and forms (rev. 5/2001). Applications must have a Dun and Bradstreet Data Universal Numbering System (DUNS) number as the Universal Identifier when applying for federal grants or cooperative agreements. The DUNS number can be obtained by calling 1-866-705-5711 or through the website at http://www.dunandbradstreet.com/. The DUNS number should be entered on line 11 of the face page of the PHS 398 form. The PHS 398 document is available at http://grants.nih.gov/grants/funding/phs398/phs398.html in an interactive format. For further assistance, contact GrantsInfo by calling 301-435-0714 or e-mailing GrantsInfo@nih.gov.

Applications submitted in response to this PA will be accepted at the following receipt dates: 13 October 2004, 13 October 2005, and 13 October 2006.

Contact: For the complete listing of contacts, please consult the full PA, available online at http://grants1.nih.gov/grants/guide/pa-files/PAR-04-116.html. Reference: PA No. PAR-04-116

